# Surgical Management of Combined Patellofemoral and Proximal Tibiofibular Joint Instability in a Patient With Ehlers-Danlos Syndrome

**DOI:** 10.1016/j.eats.2025.103902

**Published:** 2025-10-09

**Authors:** Sebastian Schmidt, Chilan B.G. Leite, Domenico Franco, Omar Protzuk, Nathan Sherman, Christian Lattermann

**Affiliations:** a Department of Orthopedic Surgery, Brigham and Women’s Hospital, Harvard Medical School, Boston, Massachusetts, U.S.A.; b Department of Orthopaedic and Trauma Surgery, University Medical Centre Mannheim, Medical Faculty Mannheim, University of Heidelberg, Mannheim, Germany; c Operative Research Unit of Orthopaedic and Trauma Surgery, Fondazione Policlinico Universitario Campus Bio-Medico, Rome, Italy

## Abstract

Ehlers-Danlos syndrome often presents with patellofemoral instability and, less commonly, proximal tibiofibular joint (PTFJ) instability, leading to compounded symptoms and functional impairment. This Technical Note describes a combined surgical approach for managing dual instability in patients with Ehlers-Danlos syndrome, involving medial patellofemoral ligament reconstruction and tibial tubercle osteotomy, in conjunction with PTFJ stabilization using an adjustable-loop cortical suspension device. This technique addresses biomechanical abnormalities and improves joint stability. Postoperative rehabilitation emphasizes controlled mobilization and progressive weightbearing. This comprehensive approach optimizes functional outcomes in patients with complex patellofemoral instability and PTFJ.

Ehlers-Danlos syndrome comprises a group of inherited connective tissue disorders characterized by joint hypermobility, ligamentous laxity, and recurrent instability.^1^, ^2^, ^3^ Hypermobile Ehlers-Danlos syndrome (hEDS) is the most prevalent subtype and is frequently associated with musculoskeletal complications, particularly patellofemoral instability (PFI), which affects over half of patients with hEDS.^4^^,^^5^ This instability results from both systemic connective tissue abnormalities and joint-specific biomechanical issues, leading to pain and functional limitations.^5^ Although PFI is well documented, instability of the proximal tibiofibular joint (PTFJ) is less commonly recognized but can significantly contribute to symptoms.^6^

When both PFI and PTFJ instability occur together, patients may experience compounded symptoms and functional impairment. Gait alterations from PFI can overload the fibular head, aggravating PTFJ symptoms, while PTFJ instability may increase lateral stress on the patella, worsening maltracking.^7^ This highlights the necessity for thorough evaluation and tailored treatment strategies.

Surgical management of PFI often includes medial patellofemoral ligament reconstruction (MPFLR), which can be combined with tibial tubercle osteotomy (TTO) in the presence of anatomic risk factors such as patella alta or elevated tibial tuberosity–trochlear groove (TT-TG) distance.^8^^,^^9^ This combined approach has been shown to reduce recurrence and improve outcomes.^8^, ^9^, ^10^, ^11^ In contrast, PTFJ instability can be treated with either ligament reconstruction or stabilization using a screw or an adjustable-loop cortical suspension device.^12^, ^13^, ^14^, ^15^ These techniques maintain joint biomechanics and reduce the risk of complications associated with screw fixation.^12^, ^13^, ^14^ The described technique integrates MPFLR, TTO, and PTFJ stabilization using a suspensory fixation device to address the complex pathology in patients with combined joint instability.

## Surgical Technique

### Patient Evaluation

PTFJ instability typically presents with lateral knee pain, subluxation during deep flexion or squatting, and possibly peroneal nerve irritation.^12^^,^^16^ In contrast, PFI is associated with anterior knee pain, patellar maltracking, and episodes of dislocation, often during early flexion activities like stair climbing.^17^ A thorough physical examination should assess patellar dynamic tracking and dynamic J-sign, and a ligamentous examination should explore the patellofemoral joint recording lateral displacement (quadrant method), PTFJ ligament integrity, and peroneal nerve involvement. Imaging plays a key role in diagnosis. Ultrasound may be used initially for evaluating medial patellofemoral ligament (MPFL) injuries or PTFJ widening, but magnetic resonance imaging is the gold standard. Magnetic resonance imaging allows comprehensive assessment of soft tissue structures, cartilage, and ligaments—including the MPFL, lateral collateral ligament, and biceps femoris tendon—and evaluates anatomic risk factors such as trochlear dysplasia, patellar height, TT-TG distance, and patellar tilt.^18^^,^^19^ Weight-bearing x-rays are essential for identifying osteoarthritic changes, mechanical axis deviations, and alignment abnormalities, which are critical in PTFJ assessment. Surgical intervention with MPFLR, TTO, and PTFJ stabilization is considered after failed conservative management or prior unsuccessful procedures. Relative contraindications include inflammatory arthritis, active infection, morbid obesity, and smoking. Preoperative exclusion of sagittal or coronal malalignment is necessary to ensure appropriate patient selection.

### Patient Position

The patient is positioned supine on a flat table with the ability to flex the foot portion. A tourniquet is positioned proximally on the thigh, and the leg is positioned into an arthroscopic leg holder. This facilitates intraoperative fluoroscopic assessment of the femoral tunnel placement (Schoettle’s point [or zone]) for the MPFLR (Video 1) as well as the PTFJ. The foot can be positioned on a cushioned Mayo stand to allow all-around access and positioning for the TTO.

### Tibial Tubercle Osteotomy

**Fig 1:**
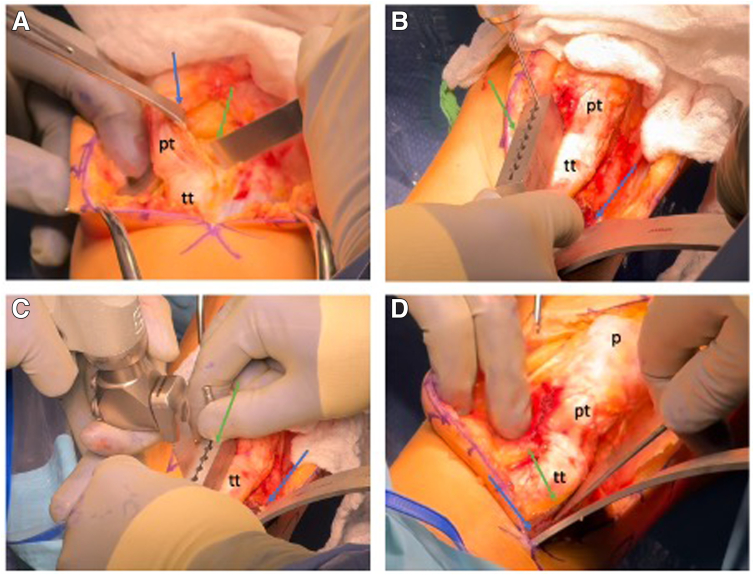
Tibial tubercle osteotomy. (A) Under direct visualization of the left knee, the patella tendon (pt) with its medial and lateral borders and the tibial tubercle (tt) are identified. A retractor (blue arrow) exposes the lateral side and a transverse cut is made just above the tibial tubercle using an osteotome (green arrow). (B) After exposing the anterolateral tibial compartment, a retractor is then slid posteriorly to the tibia (blue arrow). The cutting block (green arrow) is placed at the intersection of the osteotomy cuts, with the foot in a neutral position to guide proper osteotomy orientation. Its angle can be adjusted distally to modify the tubercle fragment size. (C) The osteotomy is initiated using a saw (green arrow), and the superior portion is intentionally left uncut to prevent the blade from directing toward the articular surface. A retractor (blue arrow) is essential during this step to protect the surrounding soft tissues. (D) With the retractor in place (blue arrow), the osteotomy is completed proximally and distally using an osteotome (green arrow).

A longitudinal midline incision is made just lateral to the patella, and subcutaneous tissues are carefully dissected. Self-retaining Gelphi retractors are positioned to allow adequate exposure of the extensor mechanism. The medial and lateral margins of the patellar tendon are identified, and electrocautery is used to dissect laterally along the tendon to the anterior proximal tibia to isolate the tibial tubercle. The medial side is similarly exposed, and the infrapatellar fat pad is partially resected to improve visualization of the true insertion of the patella tendon into the tubercle (Fig 1A).

Initially, a transverse osteotomy cut is made just proximal to the tibial tubercle, skirting the patella tendon insertion into the tubercle, followed by two 45° oblique scores extending distally on each side to define the osteotomy fragment and provide a mark for the positioning of the cutting block. The proximal anterolateral compartment is accessed via electrocautery, and its musculature is gently elevated using a periosteal elevator. To safeguard the neurovascular structures, a wide retractor (AMZ retractor; DePuy Mitek) is positioned posterior to the tibia, and the muscle is retracted laterally. The cutting block is fixed using a drill that is positioned at the intersection of the proximal tubercle cut and the medial score. The cutting block is initially aligned straight anterior/posterior (confirm with foot position), then rotated laterally using the drill as a hinge and positioned such that the tubercle fragment is about 5 to 7 cm long (Fig 1B). The angle of the cutting block can be modified to control the osteotomy angle (based upon the required medialization and anteriorization), as well as the required osteotomy size, avoiding the tibial isthmus and minimizing fracture propagation risk.

The osteotomy is started with a saw and completed using an osteotome, preserving the superior aspect of the tibia, to protect the joint surface and the lateral tibial corner (Fig 1 C and D). Soft tissue bridges are then released.

Osteotomy angle dictated tubercle translation: transverse cuts allow medialization, vertical cuts enable anteriorization, and the tubercle can be distalized along the cut as required.

### Lateral Retinacular Lengthening

To maintain vascular supply, retinacular vessels located at proximal and distal margins of the patella should be preserved.

**Fig 2:**
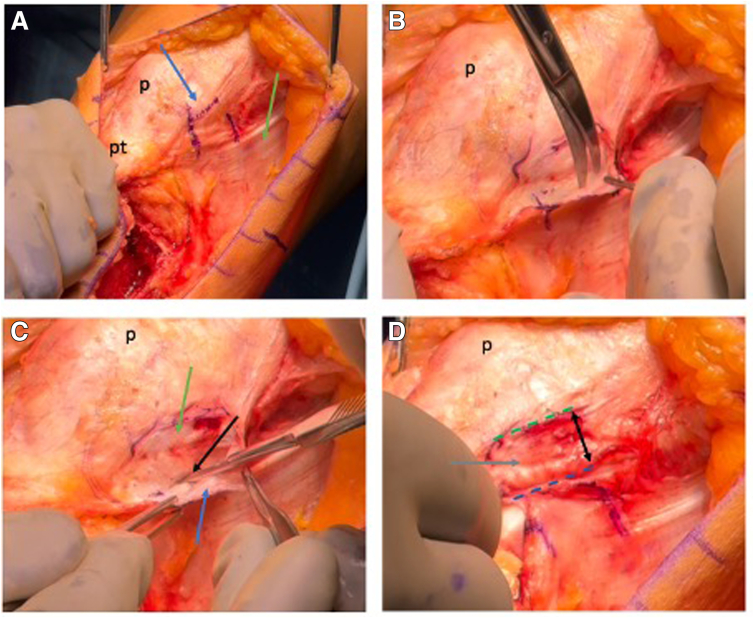
Lateral retinacular lengthening. (A) Under direct visualization of the anterolateral left knee, the lateral border of the patella (p; blue arrow), the iliotibial band (green arrow), and the lateral retinaculum are identified, with the retinaculum visualized using a marking pen. Retinacular vessels at their proximal and distal margins are carefully preserved to maintain patellar and soft tissue perfusion (pt, patella tendon). (B) After careful incision of the superficial layer, the lateral retinaculum, located within the second layer of the lateral knee structures, is dissected. (C) Following thorough dissection, the superficial lateral retinaculum (blue arrow) is elevated from the underlying deep transverse fiber layer (green arrow) after being incised 1 to 2 cm away from its patellar attachment (black arrow). (D) The superficial lateral retinaculum can be sutured to the deep transverse and capsular layer, allowing for a controlled lengthening of approximately 1 to 2 cm (distance between green and blue line; black double arrow). This adjustment can be fine-tuned during closure based on the desired tension.

After identifying the patella borders, a No. 15 blade is used to incise the upper two-thirds of the patellar fascia (Fig 2A). The lateral soft tissue complex, including the vastus lateralis, lateral patellofemoral ligament, and lateral patellotibial ligament, is dissected using Metzenbaum scissors (Fig 2B). A 1- to 2-cm incision is made laterally, parallel to the patellar border, lifting the retinaculum from the capsule. A lateral parapatellar arthrotomy is then performed.

### MPFLR and TTO Fixation

**Fig 3:**
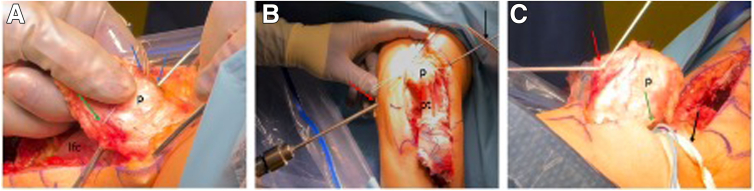
MPFL reconstruction. (A) Under direct anterior visualization of the left knee, the 9- and 11-o’clock positions (blue arrows) of the medial patella (p) are identified. A small bone trough is created, followed by 2 transpatellar drill holes (blue arrows) using a 2.5-mm drill. Two 2-0 Vicryl (Ethicon) sutures (green arrow) are then passed through as pull-through sutures (lfc, lateral femur condyle). (B) A skin incision (red arrow) is made over the medial femoral condyle, and Schöttle’s point is identified using fluoroscopy. A metal pin is positioned at this location, advanced across the femur and secured with a needle holder (black arrow). A 7-mm reamer is then used to create a unicortical femoral tunnel (pt, patella tendon). (C) The tendon graft sutures are passed through the patellar tunnel, docking the graft into the medial patella (p, green arrow) and securing it over a lateral patella bone bridge (red arrow). The graft (black arrow) is then routed through the soft tissue tunnel between layers 2 and 3 using an ultratape.

The medial side of the patella is exposed down to bone between 9- and 11-o’clock (1- and 3-o’clock on a right knee) without entering the joint. A small trough is created using a rongeur to allow for bony integration of the tendon to the patella. Two 2.5-mm transpatellar tunnels are drilled, and Vicryl (Ethicon) sutures are used to shuttle the tendon graft sutures through the patella, thus docking the tendon ends into the bony trough medially (Fig 3A). These sutures are tied laterally over a bony bridge. The resulting knot should be sunk under soft tissues, as it can sometimes be palpable.

**Fig 4:**
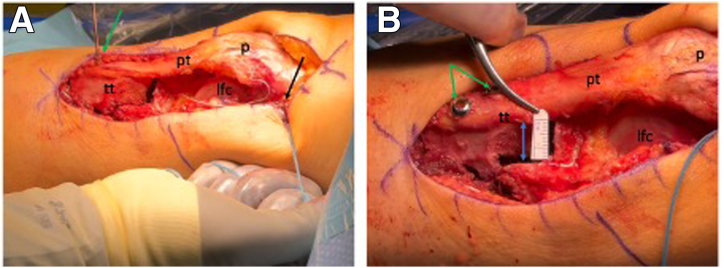
Patella tracking and tibial tubercle fixation. (A) Under direct visualization of the anterolateral left knee, a provisional fixation (green arrow) of the tibial tubercle (tt) is used to evaluate patellar tracking from extension to 120° of flexion. The medial patellofemoral ligament graft is also held under tension (black arrow) to assess stability before final fixation. (B) The tibial tubercle (tt) is fixed in the planned position using two 6.5-mm cancellous lag screws (green arrows). Screw length is confirmed under fluoroscopy. The tubercle is shifted 15 mm medially (blue double arrow) to achieve a target tibial tuberosity–trochlear groove distance of approximately 5 mm.

With the knee at 90° of flexion, Schöttle’s point is identified under fluoroscopy. A Beath pin is drilled across the medial femoral condyle, followed by a 7-mm Acorn reamer to create a unicortical femoral tunnel (Fig 3B). The graft is routed beneath layer 2 to the femoral tunnel and pulled into position using a shuttle suture. The tubercle is medialized (by 15 mm in this case) and provisionally fixed with a Steinman pin (Fig 4A). In this case, the tubercle fragment is positioned more anteriorly on the tibia, requiring the creation of a small channel on the back side of the tubercle fragment in order to seat well (Fig 4B). Stable patellar tracking is confirmed through 120° of knee flexion. Then, the tubercle is secured using two 6.5-mm partially threaded cancellous screws. Then the MPFL graft is again fully tensioned, and the knee is moved 0° to 120° a couple of times in order to set the correct length of the MPFL graft. Then, a 7 × 25-mm bioabsorbable RCI screw (Smith & Nephew) is placed into the femoral tunnel over a guidewire to secure the femoral end of the graft. Final stability is verified by confirming a reduced 1 to 2 lateral quadrant excursion at 0 and easily obtainable flexion to 120° as well as unimpeded tracking in the trochlear groove.

### Proximal Tibiofibular Joint Stabilization

**Fig 5:**
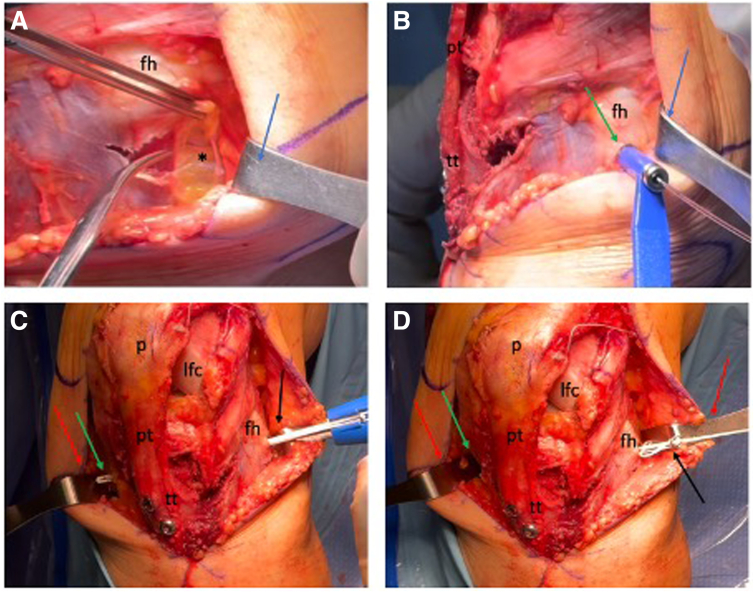
Proximal tibiofibular joint stabilization. (A) Under direct visualization of the lateral left knee in 90° of flexion, the peroneal nerve (∗) is carefully exposed and protected, with retractors used to hold surrounding soft tissue away (blue arrow). The proximal fibular head (fh) is then identified just distal to the lateral collateral ligament insertion. (B) A guidewire (green arrow) is positioned across the joint, while retracting the soft tissue (blue arrow), and the position is checked under fluoroscopy. (C) The appropriate tunnel is drilled over the guidewire, and the TightRope (Arthrex; black arrow) is inserted. On the medial side, a retractor is used to hold soft tissue aside (red arrow), allowing direct visualization of the cortical button during placement (green arrow). (D) After the cortical button is flipped (green arrow), the TightRope is tightened (black arrow), resulting in a stable proximal tibiofibular joint. The final position is confirmed under fluoroscopy.

The fibula head is easily identified through the already made lateral-based parapatellar approach. With the knee flexed to 90°, the lateral fibula head is exposed, and the peroneal nerve is identified (typically crossing the fibula neck about 3-5 cm distal to the fibular tip; Fig 5A). The nerve, once identified, can be marked and protected using a vessel loop. A guidewire is placed across the proximal tibiofibular joint, confirmed by fluoroscopy. The tunnel is placed truly perpendicular to the PTFJ orientation, which is at about 30° to 45° degrees ascending in the anteroposterior plane and 30° to 45° rotated posteriorly in the sagittal plane (Fig 5B). Hence, the tightrope drill should be slightly ascending and slightly oriented in a posterior-to-anterior direction. This positioning is important in order to avoid the anterior or posterior displacement of the proximal fibula in the PTFJ when the TightRope (Arthrex) is tightened. After tunnel drilling, the TightRope (Arthrex) is deployed (Fig 5C). The stability of the PTFJ is assessed prior to tightening of the TightRope. Then, the TightRope is progressively tightened to stabilize the joint (Fig 5D). Final positioning and stability are confirmed fluoroscopically.

### Closure

**Fig 6:**
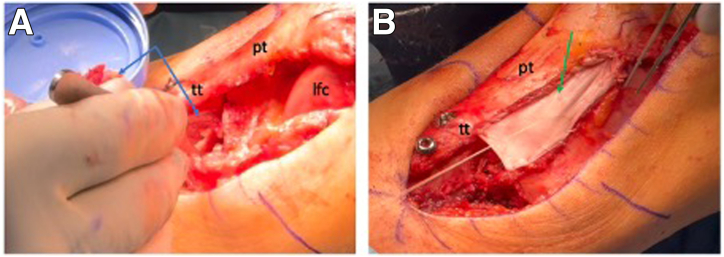
Bone grafting and capsular closure. (A) Under direct anterolateral visualization of the left knee, bone harvested from the tibial tubercle (tt) is used to graft the gap created by the cut laterally to the tibial tubercle (blue arrows). (B) To prevent lateral overtensioning following medialization, the capsule is reinforced with a Bio-Gide (Geistlich) membrane (green arrow) during closure.

In order to close the lateral soft tissues after a medialization of more than 10 mm, it is often necessary to reinforce the lateral soft tissue bridge, as the medialization does not allow for a primary closure of the lateral soft tissues. The lateral retinaculum can usually be closed after the lengthening procedure, and the capsule is reinforced using a Bio-Gide collagen membrane (Geistlich). Harvested tibial bone is used to graft the gap created by the cut laterally to the tibial tubercle (Fig 6A). The wound is irrigated, and layered closure is performed with intracutaneous skin sutures.

### Postoperative Rehabilitation

Postoperatively, patients receive deep vein thrombosis prophylaxis and use a hinged knee brace locked in extension for 2 weeks, with toe-touch weightbearing on crutches for 4 weeks. Continuous passive motion can be used and begins immediately, starting at 0° to 30° and increasing 5° daily. Weightbearing progresses after 4 weeks. Running is allowed at 4 months, low-impact activities at 6 to 9 months, and high-impact at 12 months.

## Discussion

MPFLR combined with TTO yields superior outcomes in patients with PFI compared to MPFLR alone if the TT-TG is elevated.^9^^,^^10^ Using allograft tendons for MPFLR yields similar redislocation and satisfaction rates compared to autografts but significantly reduces donor site morbidity and should be preferred for patients with connective tissue disorders such as hEDS.^20^^,^^21^ While TTO can correct patellar tracking through anteriorization, medialization, and distalization, a recent study by Varady et al.^22^ noted that anteriorization is often less than planned, requiring precise intraoperative adjustments. In contrast, medialization and distalization are more reliable.^23^^,^^24^

In some cases, patients may also present with severe trochlear dysplasia. There is growing evidence that MPFLR combined with TTO may not adequately correct patellofemoral malalignment in patients with severe dysplastic trochlea. Therefore, trochleoplasty should be considered with MPFLR and TTO in those severe cases.^25^^,^^26^

Another critical step for successful patellofemoral realignment procedures is to avoid lateral patellofemoral tightness or laxity.^27^ Lateral retinacular lengthening is performed in the subset of PFI combined with MPFLR and concomitant bony realignment procedures. Lateral retinacular lengthening outperforms lateral release, especially with tibial tubercle medialization, by reducing lateral overtensioning and medial instability risks, as well as enhancing patellar tracking control.^28^

Regarding PTFJ instability, various treatment options have been described.^12^ Although data regarding outcomes are limited, anatomic reconstruction using suspensory fixation devices should be preferred over rigid fixation, as it eliminates PTFJ micromotion, avoiding hardware complications, including screw breakage, and the need for tendon grafts, which simplifies surgery and minimizes morbidity (Tables 1 and 2). A recent biomechanical study found that a single-button suspensory fixation sufficiently stabilizes the PTFJ in case of instability.^14^ In conclusion, this combined approach addresses the complex biomechanics of PFI and PTFJ instability, optimizing functional outcomes while potentially minimizing complications.

**Table 1:** Advantages and Disadvantages

Advantages	Disadvantages
Lower risk of recurrent patellar instability compared to isolated MPFL reconstruction	Increased surgical time and complexity (multiple incisions and steps)
Single rehabilitation period instead of multiple staged surgeries	Technically demanding; requires expertise in multiple techniques and careful intraoperative planning
May restore normal knee biomechanics, improving stability and movement	Need for balanced rehabilitation (protect osteotomy while mobilizing soft tissues); possibly slower short-term functional recovery
Suspensory fixation minimizes risk of hardware irritation by using low-profile implants with a well-tolerated cortical button	Limited long-term data

MPFL, medial patellofemoral ligament.

**Table 2:** Pearls and Pitfalls

**Pearls**
• Use intraoperative fluoroscopy to ensure proper tunnel/screw trajectories (e.g., patellar sockets centered in cancellous bone). • Decompress and protect the common peroneal nerve during lateral knee exposure for PTFJ stabilization. • Use a collagen membrane (Bio-Gide; Geistlich) to cover capsular defects instead of tight capsular closure.
**Pitfalls**
• Incorrect sequencing of TTO, MPFL, and PTFJ fixation can lead to malalignment or graft overtension. • Insufficient TTO fixation or premature weightbearing can cause osteotomy nonunion or fragment displacement. • Overtightening the MPFL graft can limit knee flexion and cause medial patellar tilt or overload.

MPFL, medial patellofemoral ligament; PTFJ, proximal tibiofibular joint; TTO, tibial tubercle osteotomy.

## Declaration of Generative AI and AI-Assisted Technologies in the Writing Process

During the preparation of this work, the author(s) used ChatGPT 4o in order to improve grammar and style. After using this tool/service, the author(s) reviewed and edited the content as needed and take(s) full responsibility for the content of the publication.

## Disclosures

The authors declare the following financial interests/personal relationships which may be considered as potential competing interests: N.S. reports a relationship with *Arthroscopy* that includes editorial board membership. All other authors (S.S., C.B.G.L., D.F., O.P., C.L.) declare that they have no known competing financial interests or personal relationships that could have appeared to influence the work reported in this paper.

## Supplementary Data

Video 1 Surgical management of combined patellofemoral and proximal tibiofibular joint instability using medial patellofemoral ligament reconstruction, tibial tubercle osteotomy, and proximal tibiofibular joint stabilization.
